# Mississippi Valley Dental Society

**Published:** 1865

**Authors:** 


### MISSISSIPPI VALLEY DENTAL SOCIETY.

This, the oldest existing dental society in the world, held its twenty-first annual meeting on the 22d, 23d and 24th of February. It was the largest meeting of this society that has ever been held; and never before, perhaps, was there as much interest elicited at any of its meetings. This number contains a very full report of the discussions; but even this will exhibit, very feebly, the interest felt and manifested in the discussions and proceedings. Never before have we witnessed such enthusiasm as was then shown in reference to the subject of dental education. There was but one sentiment on the subject. All conceded there has been a great deficiency in the matter; that we have all fallen far short of our duty; that many of us have scarcely had a conception of what it ought to be, and that in the future there must be a reformation. We must study to ascertain what is our duty, and then perform it.

There was no essay on the subject of anæsthetics for the prize proffered one year ago. The silver medal offered for the most valuable invention or improvement pertaining to dental practice within the year was awarded to Dr. C. H. James, of this city, for improved vulcanizer, in which ground joints are substituted for the packing in all other machines. There were many other new and useful appliances exhibited to the society, among which we may mention three or four forms of automatic pluggers, viz, one by Dr. Baxter, a very simple and effective instrument. He has two forms of it—the one is operated by a spring, and the other by the finger. The latter the Doctor has used for several years, and has attained great facility with it. Also one by Dr. Dean, of Chicago; it is operated by a spring, upon the same general principle as Dr. B.'s. There was also one by Dr. G. Jackson, of Winona, Minn.; it is a very ingenious instrument, quite complicated in structure. In many cases, we doubt not, it will work well. In the use of all such things, practice and familiarity secure efficiency.

There were quite a number of other appliances exhibited, mention of which is made in the proceedings. There were several premiums offered for the coming year for valuable improvements, &c. This society has lost none of its vigor by age; and we hope it may flourish and be instrumental for great good to the profession so long as such agencies are needed.

